# Self-Reported Sleep Quality Across Age Modulates Resting-State Functional Connectivity in Limbic and Fronto-Temporo-Parietal Networks: An Exploratory Cross-Sectional fMRI Study

**DOI:** 10.3389/fnagi.2022.806374

**Published:** 2022-02-07

**Authors:** Giovanni Federico, Vincenzo Alfano, Federica Garramone, Giulia Mele, Marco Salvatore, Marco Aiello, Carlo Cavaliere

**Affiliations:** Istituto di Ricovero e Cura a Carattere Scientifico (IRCCS) Synlab SDN, Naples, Italy

**Keywords:** subjective sleep quality, functional connectivity, anxiety, depression, working memory

## Abstract

Sleep problems are increasingly present in the general population at any age, and they are frequently concurrent with—or predictive of—memory disturbances, anxiety, and depression. In this exploratory cross-sectional study, 54 healthy participants recruited in Naples (Italy; 23 females; mean age = 37.1 years, range = 20–68) completed the Pittsburgh Sleep Quality Index (PSQI) and a neurocognitive assessment concerning both verbal and visuospatial working memory as well as subjective measures of anxiety and depression. Then, 3T fMRI images with structural and resting-state functional sequences were acquired. A whole-brain seed-to-seed functional connectivity (FC) analysis was conducted by contrasting good (PSQI score <5) vs. bad (PSQI score ≥5) sleepers. Results highlighted FC differences in limbic and fronto-temporo-parietal brain areas. Also, bad sleepers showed an anxious/depressive behavioural phenotype and performed worse than good sleepers at visuospatial working-memory tasks. These findings may help to reveal the effects of sleep quality on daily-life cognitive functioning and further elucidate pathophysiological mechanisms of sleep disorders.

## Introduction

Sleep is a universal biological phenomenon among vertebrates that appear to be elemental to survival (Hartse, 2011). Individuals spend approximately a third of their lives sleeping, which testifies how crucial sleep is (Carlson, 2012). Even if we are far away from a complete understanding of the mechanisms underlying such complex behaviour, research has highlighted different functions of sleep that concern both cognition and emotion (Walker, 2009; Goldstein and Walker, 2014). Sleep is a homeostatic process that serves the brain’s vital functions, thus supporting cognitive activities (Mander et al., 2016). In fact, in a seemingly counterintuitive way, sleep does not appear to allow the *body* to rest, but instead, it expressly permits the *brain* to rest by reducing its metabolism (Reimund, 1994; Carlson, 2012). These aspects are crucial for individuals’ psychological functioning as sleep supports different cognitive processes involved in daily functioning (Waters and Bucks, 2011). For instance, sleep duration has been linked to verbal fluency and list memory, with both long and short sleepers having poorer performance in such kinds of tasks (Kronholm et al., 2009). Sleeping less than 5 h a night has been correlated with poorer global cognition and poorer performance in verbal memory, verbal fluency, and working memory (Tworoger et al., 2006). Sleep seems to modulate working memory performance (Chee and Choo, 2004; Kuriyama et al., 2008), namely a set of cognitive processes which provide a temporary buffer for maintaining and manipulating information, thus enabling daily cognitive tasks, such as language, learning and reasoning abilities (Baddeley, 1992). It seems that even short-term total sleep deprivation may produce effects on cognitive abilities mediated by the prefrontal cortex, which include attention and working memory (Lim and Dinges, 2010).

As a general practice derived from clinical neuropsychology, researchers investigated sleep from the systematic study of its absence, i.e., by considering sleep deprivation. Indeed, sleep deprivation impairs cerebral functioning, thus producing (1) increasing difficulties in performing cognitive tasks that require concentration, (2) perceptual distortions and, in rare cases, (3) mild hallucinations (e.g., Babkoff et al., 1989; Petrovsky et al., 2014; Sil’kis, 2014; Waters et al., 2018). Most importantly, the effects of sleep disturbances intervene rapidly as even momentary sleep perturbations may impact the individuals’ responsivity during the wake (e.g., Buzsáki and Watson, 2012). Alongside adverse effects on cognitive performance, disturbed sleep has been associated with poor emotional and physical health, substance use, conduct problems, anxiety, and depression (Reimer and Flemons, 2003; Holley et al., 2011; Alvaro et al., 2013; Conroy and Arnedt, 2014). Sleep disorders, particularly insomnia, are increasingly important problems nowadays, with incidences that grow with age (e.g., Foley et al., 1999; Gooneratne and Vitiello, 2014; Gulia and Kumar, 2018). In the general population, these disorders may relate to many factors, for instance, the use of caffeine, tobacco, alcohol, bad sleep habits (i.e., working late, using computers and other electronic devices, watching TV late at night), and modern society’s “24/7” rhythm (e.g., Brunborg et al., 2011; Poceta and Mitler, 2013; Garcia and Salloum, 2015). What is more, in addition to the endogenous and exogenous factors we reported above, sleep disorders may also emerge physiologically as age increases since variations in sleep patterns constitute part of the normal ageing process (e.g., Gulia and Kumar, 2018).

The evidence we briefly described above shows how sleep, on the one hand, may support cognitive and affective functioning and, on the other, may be susceptible to physiological and pathological favoured-by-ageing deterioration. Therefore, most studies focused on specific clinical populations or definite age clusters. However, a certain degree of sleep-related problems may also be found in the general population without established sleep disorders (e.g., Broman et al., 1996; Ohayon, 2011). Such problems may be concurrent with, or predictive of, memory disturbances, anxiety and depression, hence suggesting that sleep quality can be seen as a multidimensional and transdiagnostic variable in maintaining bio-psychological wellbeing (Lemola et al., 2013). Thus, studying the impact of sleep quality in healthy populations may help disentangle the relations between sleep, memory, ageing, and psychopathology, hence elucidating the effects of sleep on daily-life cognitive functioning. To examine the relations between sleep and changes in brain structure and function, over the years, multiple fMRI techniques, including resting-state fMRI, have been devised (e.g., Desseilles et al., 2008; Spiegelhalder et al., 2015; Cavaliere et al., 2020). On that basis, in this exploratory cross-sectional study, we investigated the effects of self-reported sleep quality on functional brain connectivity (FC), assessed by using 3T resting-state fMRI. We collected data from a sample of participants of varying ages (20–68 years). Behaviourally, we examined the effect of self-reported sleep quality on a neurocognitive process involved in daily-life cognitive functioning, namely working memory. We used the Italian version of the Pittsburgh Sleep Quality Index (PSQI) as a standardised self-report questionnaire to assess participants’ subjective sleep quality (Buysse et al., 1989; Curcio et al., 2013). In addition, we included subjective measures of anxiety and depression to characterise participants’ neuropsychological phenotype further.

## Methods

### Participants

We enrolled a convenience sample including 54 healthy participants (23 females; mean age = 37.1 years, range = 20–68). The cohort of the study came from a more extensive study, part of a research project of the IRCCS Synlab SDN (Naples, Italy). All participants were recruited in Naples (Italy) according to the following inclusion criteria: (i) lack of current or past history of alcohol or drug abuse; (ii) lack of current or past history of major psychiatric or neurological illnesses; and (iii) lack of current or past use of psychoactive medications. Both an experienced neurologist and neuropsychologist assessed each participant before starting the experiment. Participants with incidental brain lesions to the MRI examination were excluded. No participants were excluded according to the inclusion criteria, clinical evaluations, and MRI artefacts. Each participant provided written informed consent to participate in the study. The local Ethics Committee has approved the study as all its procedures followed the ethical standards laid down in the 1964 Helsinki Declaration.

### Materials

To conduct the neuropsychological assessment, several pencil-and-paper tools were used. The Italian version of the Pittsburgh Sleep Quality Index (PSQI; Buysse et al., 1989; Curcio et al., 2013) has been used to measure participants’ self-reported sleep quality. PSQI is a self-report subjective sleep-quality index that scores from 0 to 21, with a cut-off score of 5, which discriminates between good (PSQI score <5) and bad sleepers (PSQI score ≥5). The Italian version of PSQI is “a useful, valid, and reliable tool for the assessment of sleep quality, with an overall efficiency comparable to the mother language version and differentiates “good” from “bad” sleepers. The Italian version of the questionnaire provides a good and reliable differentiation between normal and pathological groups, with higher scores reported by people characterised by impaired objectively evaluated sleep quality” (Curcio et al., 2013, p. 511). Beck Depression Inventory (BDI-II; Beck et al., 1996) has been used to subjectively measure depression score (score range: 0–63; cut-off value for mild depression: 20). State-Trait Anxiety Inventory (STAI; Spielberger et al., 1983) has been used to measure trait and state anxiety. STAI consists of 20 items that assess trait anxiety (STAI-T) and 20 items that assess state anxiety (STAI-S). The Digit Span Forward and Backward tests (DSF and DSB, respectively) have been used to investigate brief-term verbal memory and working memory’s verbal component. Finally, the Corsi Block-Tapping test (CBT) has been used to assess visuospatial working memory.

### Procedure

This study has been conducted at the IRCCS Synlab SDN (Naples, Italy). An expert neuropsychologist performed the neuropsychological assessment for each participant. Such an assessment included a self-report evaluation of subjective sleep quality (PSQI), an assessment of depression and anxiety, and an evaluation of verbal and visuospatial working memory performance. After the neuropsychological assessment, each participant underwent a resting-state fMRI experimental protocol. MR images were acquired using a Biograph mMR 3T scanner (Siemens Healthcare, Erlangen, Germany) and a 12-channel head coil. An *ad hoc* acquisition protocol was devised, which included the following structural and functional sequences: (1) 3D T1-Magnetisation Prepared Rapid Acquisition Gradient Echo (MPRAGE), voxel size 0.8 × 0.8 × 0.8 mm^3^, Field of View (FOV) 214 × 214 mm, TR/TE/TI = 2,400/2.25/1,000 ms, scan time 5:03; and (2) Resting-state fMRI, Echo Planar Imaging-Gradient Echo sequence (EPI-GRE), voxel-size 4 × 4 × 4 mm^3^, TR/TE = 1,000/21.4 ms, 350 measurements, bandwidth: 2,230 Hz, scan time 6:02.

### fMRI Data Analyses

fMRI data were analysed with Functional Connectivity Toolbox (CONN v. 20b; Whitfield-Gabrieli and Nieto-Castanon, 2012; Alfano et al., 2021) and Statistical Parametric Mapping (SPM v. 12). Both CONN and SPM were executed on MATLAB (v. 2021b). With CONN, pre-processing was carried out using a pipeline that includes realignment, slice-timing, functional-image normalisation in the Montreal Neurological Institute (MNI) space, outlier detection with ART-based scrubbing smoothing, and physiological denoising. We conducted a first statistical analysis to assess participants’ resting-state brain activations. Then, we devised a second statistical data analysis to assess differences in functional connectivity (FC) between participants with higher PSQI scores (i.e., bad sleepers) and participants with lower PSQI total scores (i.e., good sleepers). We evaluated FC differences between these two sub-groups by performing CONN-based seed-to-seed analyses, which were conducted by adopting cortical and subcortical ROIs (FSL Harvard-Oxford maximum likelihood cortical and subcortical atlas, dividing bilateral areas into left/right hemisphere for a total of 106 ROIs). Finally, a third, regression-based, between-network FC analysis was conducted to investigate correlations between large-scale brain networks FC and PSQI scores (networks from CONN’s ICA analyses of HCP dataset for a total of eight networks with 32 subnetwork ROIs). For both the seed-to-seed and network-to-network comparisons, an alpha level of 0.05 was used with false discovery rate (FDR) correction for multiple comparisons (Benjamini and Hochberg, 1995).

### Behavioural Data Analyses

Pearson correlations were calculated among the PSQI scores, age, and other scores at the neuropsychological tests (i.e., STAI-T, STAI-S, BDI-II, DSF, DSB, CBT). All the scores were corrected for age, gender, and education level. An alpha level of 0.05 was used for all the analyses. The jamovi package (v. 1.6.23) for R software (v. 4.0.2) was used to perform behavioural data’s statistical analyses.

## Results

### Functional Connectivity Results

**Figure 1:**
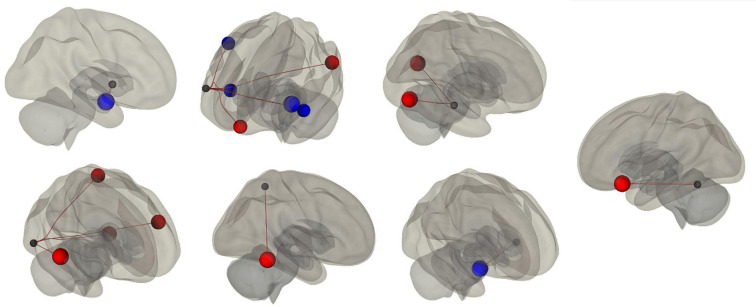
First-level functional connectivity (FC) analysis. 3D graphical representation of the first-level whole-brain seed-to-seed FC analysis’s results (Table 1), which shows the FC differences we found in bad sleepers (i.e., participants with a PSQI score ≥5) as compared to good sleepers (i.e., participants with a PSQI score <5). FC reductions are depicted in blue, while FC increases are illustrated in red.

**Figure 2:**
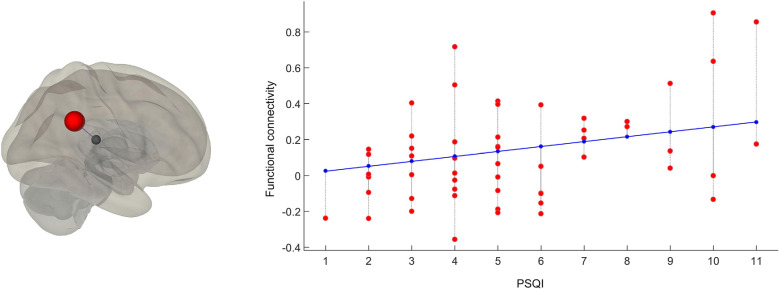
Between-network regression-based functional connectivity analysis. 3D graphical representation of the second-level, regression-based, between-network functional connectivity analysis’ results. FC hyperconnectivity between the Salience network (peak seed: right SMG; on the left in red) and the Frontoparietal network (peak seed: Posterior Parietal Cortex; on the left in blue) positively correlates with participants’ PSQI scores (scatter plot on the right; the x-axis represents the distribution of PSQI scores while the y-axis represents the functional connectivity value in the seeds).

Results of the first whole-brain seed-to-seed analysis we implemented showed significant differences between participants with higher PSQI scores (i.e., 31 bad sleepers) and participants with lower PSQI (i.e., 23 good sleepers). The following pattern of seed-to-seed hypoconnectivity was found: right insular cortex (IC)—the right amygdala (*T* = −4.0; *p* = 0.024); pars triangularis of the right inferior frontal gyrus (IFGtri)—both left (*T* = −3.8; *p* = 0.035) and right IC (*T* = −3.2 *p*; = 0.05), right anterior supramarginal gyrus (aSMG; *T* = −0.2; *p* = 0.05), and left planum polare (PP; *T* = −3.1; *p* = 0.05); and left PP—right amygdala (*T* = −4.4; *p* = 0.006). The following pattern of seed-to-seed hyperconnectivity was found: right IFGtri—left posterior middle temporal gyrus (pMTG; *T* = 3.4; *p* = 0.04), left angular gyrus (AG; *T* = 3.3; *p* = 0.05), and medial frontal cortex (MedFC; *T* = 3.3; *p* = 0.05); posterior division of the right superior temporal gyrus (pSTG)—inferior division of the left lateral occipital cortex (iLOC; *T* = 3.7; *p* = 0.027); right iLOC—right pSTG (*T* = 3.8; *p* = 0.027), temporo-occipital part of the left inferior temporal gyrus (toITG; *T* = 3.5; *p* = 0.043), left IFG (*T* = 3.4; *p* = 0.043), and left superior parietal lobule (SPL; *T* = 3.3; *p* = 0.043); left SPL—right toITG (*T* = 3.7; *p* = 0.05); and left toITG—left frontal orbital cortex (OFC; *T* = 3.5; *p* = 0.05). The second analysis assessed between-network FC by considering the PSQI score as a continuous regressor. A moderate positive correlation among the PSQI score, the Salience network (peak seed: right SMG) and the Frontoparietal network [peak seed: right Posterior Parietal Cortex (PPC); *T* = 3.4; *r* = 0.42; *p* = 0.043] was found. Results of first FC analysis are summarised in Table 1 and Figure 1, while the results of the second FC analysis are depicted in Figure 2.

**Table 1:** First-level whole-brain seed-to-seed FC analysis’s results.

High PSQI (bad sleepers) > Low PSQI (good sleepers)			
Seed	Target	T-score	p-FDR
Right Insular Cortex	Right Amygdala	4.0	0.024
Right IFGtri	Left Insular Cortex	3.8	0.035
	Left pMTG	3.4	0.04
	Left Angular Gyrus	3.3	0.05
	Frontal Medial Cortex	3.3	0.05
	Right Insular Cortex	−3.2	0.05
	Right aSMG	−3.2	0.05
	Left Planum Polare	−3.1	0.05
Right pSTG	Left iLOC	3.7	0.027
Left toITG	Left Frontal Orbital Cortex	3.5	0.05
Right iLOC	Right pSTG	3.8	0.027
	Left toITG	3.5	0.043
	Left Inferior Frontal Gyrus	3.4	0.043
	Left Superior Parietal Lobule	3.3	0.043
Left Superior Parietal Lobule	Right toITG	3.7	0.05
Left Planum Polare	Right Amygdala	−4.4	0.006

Bad sleepers (i.e., participants with a PSQI score ≥5) were compared with good sleepers (i.e., participants with a PSQI score <5). Positive T scores represent FC hyperconnectivity, while negative T scores represent FC hypoconnectivity. p-FDR, *p-value* corrected for multiple comparisons (Benjamini and Hochberg, 1995). IFGtri, pars triangularis of the inferior frontal gyrus; pMTG, posterior middle temporal gyrus; aSMG, anterior supramarginal gyrus; pSTG, posterior division of the superior temporal gyrus; iLOC, inferior division of the left lateral occipital cortex; toITG, temporo-occipital part of the inferior temporal gyrus.

### Behavioural Results

Significant positive Pearson’s correlations were found among PSQI scores and: STAI-S scores (*r* = 0.34; *p* < 0.05); STAI-T scores (*r* = 0.64; *p* < 0.001); BDI-II scores (*r* = 0.58; *p* < 0.001); and age (*r* = 0.34; *p* < 0.05). A significant negative correlation between PSQI scores and CBT scores was found (*r* = −0.36; *p* < 0.01). Two non-significant negative correlations were found among PSQI scores and: DSF (*r* = −0.14, *p* = 0.31); and DSB (*r* = −0.13, *p* = 0.37). Descriptive statistics concerning all the measures considered in the study are summarised in Table 2, while a correlation matrix of them is presented in Table 3.

**Table 2:** Descriptive data concerning all the neuropsychological tests involved in the study (i.e., STAI-T, STAI-S, BDI-II, DSF, DSB, CBT).

	**PSQI**	**Age**	**BDI-II**	**STAI-T**	**STAI-S**	**DSF**	**DSB**	**CBT**
Mean	5.35	37.1	6.63	38.2	32.2	5.59	4.20	5.56
Standard deviation	2.61	12.9	7.16	9.94	8.35	0.957	1.29	1.20
Minimum	1	20	0.00	22	20	2.77	2.26	1.57
Maximum	11	68	27.0	61	68	8.47	7.43	7.39

PSQI, Pittsburgh Sleep Quality Index score; BDI-II, Beck Depression Inventory score; STAI-T, State-Trait Anxiety Inventory (trait anxiety score); STAI-S, State-Trait Anxiety Inventory (state anxiety score); DSF, Digit Span Forward score; DSB, Digit Span Backward score; CBT, Corsi Block-Tapping score.

**Table 3:** Pearson’s correlation matrix concerning all scores at the neuropsychological tests involved in the study (i.e., STAI-T, STAI-S, BDI-II, DSF, DSB, CBT).

		**PSQI**	**STAI-S**	**STAI-T**	**BDI-II**	**CBT**	**DSF**	**DSB**
Age	Pearson’s r	0.343*						
	*p*-value	0.011
STAI-S	Pearson’s r	0.335*	—	0.637***				
	*p*-value	0.023	—	<0.001
STAI-T	Pearson’s r	0.635***		—				
	*p*-value	<0.001		—
BDI-II	Pearson’s r	0.580***	0.695***	0.795***	—			
	*p*-value	<0.001	<0.001	<0.001	—
CBT	Pearson’s r	−0.359**	−0.045	−0.282	−0.182	—	0.373**	0.274
	*p*-value	0.010	0.768	0.060	0.232	—	0.007	0.052
DSF	Pearson’s r	−0.144	−0.057	−0.181	−0.259		—	
	*p*-value	0.312	0.712	0.233	0.086	—
DSB	Pearson’s r	−0.128	−0.114	−0.172	−0.174		0.459***	—
	*p*-value	0.370	0.454	0.259	0.252	<0.001	—

Note: *p < 0.05; **p < 0.01; ***p < 0.001. PSQI, Pittsburgh Sleep Quality Index score; BDI-II, Beck Depression Inventory score; STAI-T, State-Trait Anxiety Inventory (trait anxiety score); STAI-S, State-Trait Anxiety Inventory (state anxiety score); DSF, Digit Span Forward score; DSB, Digit Span Backward score; CBT, Corsi Block-Tapping score.

## Discussion

We analysed the brain functional connectivity (FC) of a large sample of healthy participants of varying ages, taking into account the self-reported quality of their sleep according to the Pittsburgh Sleep Quality Index (PSQI; Buysse et al., 1989) and other neuropsychological measures related to anxiety (i.e., State-Trait anxiety inventory, STAI; Spielberger et al., 1983), depression (i.e., Beck Depression Inventory, BDI-II; Beck et al., 1996), verbal working memory (i.e., Forward and Backward Digit Span), and visuospatial working memory (i.e., Corsi Block-Tapping tasks). For each participant, we first carried out the brief neuropsychological assessment and then, on the same day, acquired 3T resting-state fMRI images. Given its interoperability with other high-level cognitive processes (e.g., reasoning, language comprehension, and learning), we considered participants’ working-memory-related scores as a general measure of cognitive functioning involved in daily tasks (Baddeley, 1992). Thus, on the one hand, we analysed some of the neuropsychological sequelae recurrently associated with sleep problems. On the other hand, we assessed whether, and if so how, sleep quality may modulate whole-brain FC. We did not involve a specific clinical sample, focusing on sleep problems possibly occurring in large-scale healthy populations across different ages. Our results showed that sleep quality might modulate the FC of a large set of limbic and fronto-temporo-parietal brain areas that are crucially involved in everyday cognitive functioning. Consistent with the neuroimage data, behavioural results show a neuropsychological profile characterised by higher anxiety, depression and worse cognitive performance among bad sleepers.

The main indication of this study concerns the modulation of resting-state FC as a function of participants’ self-reported sleep quality. We investigated FC by making distinct analyses at several levels of detail. As a first-level analysis, we considered the effects of self-reported sleep quality on FC by contrasting good sleepers (i.e., participants with PSQI score <5) with bad sleepers (i.e., participants with PSQI score >5). Such an analysis highlighted a diversified seed-to-seed FC modulation pattern involving multiple cortical and subcortical areas. In particular, bad sleepers exhibited FC reductions between right IC and right amygdala; between right IFGtri and both left and right IC, right aSMG, and left PP; between left PP and right amygdala. In bad sleepers, we also found FC increases between right IFG and left pMTG, left AG, and MedFC; between right pSTG and left iLOC; between right iLOC and right pSTG, left toITG, left IFG, and left SPL; between left SPL and right toITG; between left toITG and left OFC. The interplay among large-scale brain networks is critical for supporting cognitive functions since information processing in the cerebral cortex involves functional interactions among distributed brain areas (e.g., Yeo et al., 2011). Therefore, sleep-quality-related cognitive effects might reverberate on large-scale brain networks connectivity. Based on that, as a second-level analysis, we assessed between-network FC by considering PSQI scores as a distinct source of variance. Consistent with the first-level seed-to-seed analysis, we found a moderate positive correlation among the PSQI score, the Salience network (with the peak seed located in right SMG) and the Frontoparietal network (with the peak seed located in the right PPC).

We found peculiar FC modulation patterns concerning cortical and subcortical brain areas critically associated with distinct neurocognitive functions. For instance, the IC is part of the salience network, which permits the identification of behaviourally relevant stimuli in the environment, thus coordinating the allocation of attention and neural resources to them (e.g., Uddin, 2014). Extensive literature underlines how sleep may directly modulate IC activity (Flynn, 1999), and many fMRI studies highlighted the impact of sleep disturbances in altering the neural activity of IC alongside frontoparietal networks (e.g., Chee et al., 2006; Chuah et al., 2006; Venkatraman et al., 2011). Chronic sleep deprivation may negatively impact attention and alertness due to prefrontal cortex dysfunctions (Killgore, 2010). Such a “sleep-related hypofrontality” may produce waking-state instability, which may reverberate in several cognitive processes, such as working memory, visuomotor abilities and reasoning skills (Alhola and Polo-Kantola, 2007). Consistent with our behavioural results, impairments in working memory after sleep deprivation seem to be associated with reductions in prefrontal and parietal cortex activations (Mu et al., 2005). The functional hypoconnectivity we found in bad sleepers between right IFG and right SMG and the reduced performance at the working memory task seems to be rather suggestive in this respect. Activations of frontoparietal regions after normal sleep have been recently negatively correlated with working-memory performance decline from normal sleep to 24 h of sleep deprivation, differentiating individuals who maintained such performance following sleep deprivation from those who were more vulnerable to its effects (Chee et al., 2006). Thus, FC increases in different fronto-temporo-parietal regions might signal compensatory mechanisms which intervene as an effect of poor sleep quality, thereby differentiating individual tolerance to sleep deprivation. Whereas frontal lobes are particularly vulnerable to sleep disturbances, more posterior brain mechanisms might compensate for the relative deficits produced by the sleeping debt (e.g., Gosselin et al., 2005). Our results show both hyper- and hypo-activations in FC among areas of opposite hemispheres, possibly indicating how such a compensatory mechanism may involve both hemispheres. Concerning the subcortical involvement, recent evidence indicated that self-reported sleep quality might moderate the relationships among the amygdala reactivity, negative affectivity, and perceived stress (Prather et al., 2013). Therefore, limbic FC modulation and the anxious/depressive behavioural pattern we found in bad sleepers seem to support the strong connections highlighted in the literature among sleep disorders, anxiety/depressive disorders and negative affectivity (e.g., Alvaro et al., 2013; Prather et al., 2013).

By considering the behavioural data, we found multiple associations between self-reported sleep quality and the neuropsychological variables we analysed. In particular, we found positive correlations among the self-reported sleep quality score and both the anxiety scores we used (i.e., trait anxiety and state anxiety). We also found positive correlations between the self-reported sleep quality score and the depression score. In this respect, our findings are consistent with the literature, as sleep disorders have been often associated with behavioural disorders such as anxiety and depression (Alvaro et al., 2013). Indeed, about 90% of depressed individuals may complain of sleep disorders, while insomnia patients may have twice the risk of developing depression during their lifetime (Baglioni et al., 2011). It should be noted that a general dysfunctional emotional reactivity’s mechanism, which mediates the association between insomnia, depression and anxiety, has been recently proposed (Baglioni et al., 2010). Interestingly for our purposes, both anxiety and depression have been associated with attention, working memory and executive function impairments, which are neurocognitive functions mediated by the frontoparietal network (e.g., Kane and Engle, 2002; Castaneda et al., 2008; Rossi et al., 2009; Friedman and Robbins, 2022). Congruently, we found differences in the functional connectivity of these brain areas as a function of self-report sleep quality. Behaviourally, we found negative correlations between self-reported sleep quality and the visuospatial working memory’s task. Intriguingly, we found no correlations among self-reported sleep quality and verbal working memory tasks, thus suggesting sleep quality may impact working memory’s visuospatial component preferentially. It is worth noticing that right-brain networks are more involved in the visuospatial working memory than left ones, while left-brain structures are involved more than right ones in verbal working memory (Hennecke et al., 2021). Thus, spatial abilities are more related to right hemisphere activations (van Asselen et al., 2006). Consistent with that, we found a primarily right-lateralised FC hypoconnectivity in bad sleepers, which may have contributed to degrading visuospatial working memory performance.

The present study has some limitations concerning its cross-sectional nature (i.e., the inability to trace direct causal mechanisms between participants’ sleep quality and their behavioural phenotype) as well as the absence of a direct comparison between objective and subjective sleep quality’s measures (e.g., Landry et al., 2015; Hsiao et al., 2018). A third limitation concerns the convenience sample. However, *post hoc* evaluation of the literature indicated such a sample size as adequate for an exploratory fMRI study. Indeed, similar neuroimaging studies on the effects of poor sleep quality, sleep deprivation, and insomnia used even smaller samples (e.g., Bu et al., 2018; Kong et al., 2018; Li et al., 2019). Therefore, while the results we reported appear to be quite suggestive in indicating effects in the functional brain connectivity that might refer to the behavioural phenotype we found, further studies involving larger samples as well as objective measures of sleep quality should corroborate and extend the preliminary findings we presented here.

## Conclusions

The preliminary findings we report here may help reveal the effects of sleep quality on daily-life cognitive functioning and further elucidate pathophysiological mechanisms of sleep disorders. Indeed, taken as a whole, our results support a consistent trend in literature that underlines how bad sleepers may expose a neurocognitive profile characterised by higher levels of anxiety and depression and lower visuospatial working memory functioning. Furthermore, we found differences in the functional brain connectivity that may reasonably refer to such a behavioural phenotype. Alongside drawing connections between sleep quality and subsequent cognitive and psychological effects, we speculated about a supplementary mechanism that may support the cognitive function in the case of qualitative and quantitative sleep deprivation. Although the idea we present may appear plausible and recent evidence seems to support it, further experimental evidence is required.

## Data Availability Statement

The raw data supporting the conclusions of this article will be made available by the authors, without undue reservation.

## Ethics Statement

The studies involving human participants were reviewed and approved by Ethics Committee of IRCCS Pascale. The patients/participants provided their written informed consent to participate in this study.

## Author Contributions

GF, CC and VA conceived the study. GF, VA, FG and GM acquired behavioural and fMRI data and conducted the study. GF and VA analysed the data. GF wrote the manuscript’s first draft. VA, FG, GM, MA, MS and CC revised the manuscript and provided critical comments and theoretical contributions. All authors contributed to the article and approved the submitted version.

## Conflict of Interest

The authors declare that the research was conducted in the absence of any commercial or financial relationships that could be construed as a potential conflict of interest.

## Publisher’s Note

All claims expressed in this article are solely those of the authors and do not necessarily represent those of their affiliated organizations, or those of the publisher, the editors and the reviewers. Any product that may be evaluated in this article, or claim that may be made by its manufacturer, is not guaranteed or endorsed by the publisher.
